# Editorial and Miscellaneous

**Published:** 1863-12

**Authors:** 


### EDITORIAL AND MISCELLANEOUS.

*The Principles and Practice of Ophthalmic Medicine and Surgery.* By T. WHARTON JONES, F. R. S., etc. With 117 illustrations. Third American Edition, with Additions. Philadelphia. Blanchard & Lea. Vol. 3. Scott, Keen & Co., Chicago.

This work of Mr. Jones has already been noticed in the *Journal*, and has become a standard, both in England and the United States. The present edition is somewhat improved from the last, and brought fully up to to the most recent views on the subject. It ought to be in the library of every physician who treats diseases of the eye. D. B.

---

*Conservative Medicine as Applied to Hygiene.*—DR. FLINT has favored the thinking portion of the medical profession with another of his trenchant articles on "Conservative Medicine"—those principles "which lead the practitioner in dealing with diseases to preserve, develop and support the vital powers." In his last essay, in the *Am. Med. Journal*, he considers these principles as applied to Hygiene, both in health and disease. We have only space for the salient points.

"To weaken the powers of life, or impair the constitutional strength, is never the design of measures to prevent disease."

There is no such condition as an "overplus of health."

Bleeding and purging as prophylactics have no warrant in modern science. Not even gestation warrants either of them.

Restricting the diet is equally repugnant to sound philosophy either prior to surgical operations or as prophylactic of contagious diseases. Zymotic diseases do not subvert this rule.

"Wear and tear" of the mental faculties, under it may be the stimulus of the most laudable ambition, notwithstanding the individual is regular in his habits, temperate and moral, will beget disease, as witness tuberculosis, Bright's diseases, diabetes, etc. It is a dangerous egotism of the individual that he can bear an unlimited duration of work—mental or physical. The system will, sooner or later, break down under unremitted labor even though the powers are not seemingly at any one time overtaxed. It is a fallacy that mental and physical exertions counterbalance each other. The exhaustion incident to excessive mental labor is aggravated by the superaddition of muscular fatigue.

The evils of overfeeding, "gormandizing," are mental and moral rather than physical. The healthiest, ablest and best men and women have been good feeders. An excess of ingesta over the absolute wants of the economy is not necessarily an evil, there being provision made for the disposition of an overplus, but none against a deficiency of aliment. Generous living constitutes an important part of the curative management of very many diseases.

Hunger, appetite and taste are designed to govern dietetics. Watching anxiously the progress of digestion is extremely fallacious, and conduces to digestive disorder. The question to the physician: "What drugs shall I give to effect a cure?" is infinitely less important than that other: "What hygienic relations will contribute to the recovery of the patient?"

Disease does not stand in the way of patients from starvation. Deficiency of assimilation originates the symptomatic phenomena in acute diseases, to a greater or less extent, and is thus a greater or less source of danger.

It is a constant and important element. In chronic diseases it is a paramount element. The giving of about all medicines is to be guided by their influence upon the energy of the nutrient and assimilative processes. This is peculiarly true in all continued and "typhoid" fevers.

"Under all circumstances, a chronic affection is less likely to be prolonged, serious lesions of structure are less likely to take place, and a fatal termination is postponed in proportion as the vital powers are preserved." All hygienic measures are to be conjoined.

Air is a grand element of hygiene. "Free and daily exposure is important as a means of preserving the vigor of the body in health; may it not be equally important as a means of keeping up the vital powers in disease? Atmospherical vicissitudes, even in disease, there is reason to believe are hygienic. Prevailing apprehensions on the subject are much exaggerated. Mental conditions require careful management. The reciprocal agencies of mind and matter can scarcely be overrated. The resisting power to disease is largely mental as well as physical.

The emotions must be properly controlled by the physician. Confidence and hope are wonderfully potent. Prof. Flint admits the clergy as subsidiary to the office of calming the patient's mind. He has evidently been more happy than we have in this respect. To us this is a *questio vexata* still.

Hospital arrangements are too often unfavorable to proper mental hygiene. All this is being corrected in the best managed and controlled institutions.

We look anxiously for Prof. Flint's promised work on Practice, as likely to supersede wholly the Empiritical treatises now in vogue.

---

*Medical Logic.* An Introductory Lecture to the Med. Dep. of the Univ. of Mich. Session 1863–4. By S. G. Armor, M. D., Prof. of Instit. of Med. and Mat. Med.

The unknown friend who forwarded to us this philosophical brochure has our sincere thanks. We congratulate Prof. Armor on both the sound logic and common sense of his lecture, and the fact that the Detroit Medical Society no longer exists to denounce him by resolution for the promulgation of the views he entertains on the points discussed.

The relics of that old organization ought certainly to have their professional bones stirred in their coffins by the conviction expressed by the learned lecturer, "that in no department of human inquiry is there so much *bad logic* as in the investigation of questions relating to Medical Science." Witness this and lament, O Disembodied Spirit of the Detroit Medical Society!

We are all too apt to assume, as a postulate, that to be sick is to be visited by the Angel of Death; that the natural history of disease is death; that the warfare is therefore between *death* and *drugs*. With minds thus educated, *post hoc* is always *propter hoc;* and hence the quackery, the cures, the "specifics" which afflict our race. It is my firm conviction that if we could induce people to respect *Nature* more, and boastful pretenders of the healing art less, it would rid the world of a vast amount of bad logic. They would be prepared to reason from a new stand point; the fierce schisms of sects and schools would give way to Rational Medicine; we would have no occasion for another edition of Bigelow's "*Paradise of Doctors*," Nature and the Doctor would respectively occupy their true positions,—the one as the minister and handmaid of the other.

Again take this:

False and incompetent witnesses should be even more rigidly excluded from the Temple of Science than from our Temples of Justice. They invest error, for the time being, with all the power and majesty of Truth, and thus destroy the harmony which should ever exist between Truth and the highest development of our race.

By the term *evidence*, I mean to include, in a general sense, all the means by which any alleged matter of fact is established or disproved. In Medical Science, *facts*—not hypothesis—are what we are after, and this, as we shall see, is a striking characteristic of Rational Medicine as contra distinguished from all modern systems and theories. Facts, then, being the object of our research, and these being derived from others, as well as from our own observation, the importance of carefully considering the ground of our belief as based upon *reliable testimony*, becomes at once apparent. Nothing but a rigid adherence to the justly interpreted laws of evidence, will prevent us from being constantly led into error. But for the supremacy of induction, as the only reliable mode of discovering medical truth, Medical Science would forever grope her way in the dark. Induction begins with facts; deduction begins with ideas. By the former mode of reasoning, Medical Science has always made real and lasting progress; by the latter the most visionary one-idead systems have been established upon the most illogical, irrelevant, and inconclusive reasoning,—rules drawn from the most narrow and selfish views.

These errors in reasoning have their origin in the very nature anp constitution of the human mind. There is an inherent tendency to abstract and generalize, and our generalizations are too often hasty and immature.

The author avows himself an advocate of Rational Medicine.

It is mortifying to observe, in our own journals, the headlong haste with which, every day, some new remedy is taken into favor, eulogised and applauded as a catholicon for all the ills to which human flesh is heir, without caution, without selection, without discrimination, and without reference to the rules of a safe logical induction from reliable premises. All such observations, all such teachings and records, should be rigidly excluded, as unfit to form the basis of sound conclusions. * * * * * * * * *

This is the glory of Rational medical science. It has no theory to maintain, no creed to enunciate. It follows *facts*, well observed, carefully studied, and based on a sufficiently wide range of observation, let them emanate from what source they may, or militate against whose theory they may. In this it differs from all other one-idead systems of practice, which are "based on an exclusive dogmita, to the rejection of the accumulated experience of the profession." Our science, let it ever be borne in mind, does not rest on abstract medical doctrines. Doctrines, as such, we have none; theories, none; no "system," as such, to maintain; no creed to follow. Rational medical science plants itself on the notation, arrangement and classification of facts. Hence, I affirm, its votaries work in the only true direction of a safe and rational progress. Where facts lead them, there they go, unmindful of any creed, or doctrine, or school. Rational medical science is unlimited in its resources. Untrammeled by the dogmas of schools or creeds, we boast of a science and art purely and truly *Eclectic.*

He has a want also for which the *debris* of the Society aforesaid should severely censure him by resolution:

What we want,—what medical science requires above all things, is manly independence of investigation, associated with a *rational skepticism in the reception of testimony.* Gœthe hes an excellent aphorism defining that state of mind which he calls "Thatige Skepsis"—active doubt. "It is a doubt which so loves truth, that it neither dares rest in doubt, nor extenguish itself by unjustified belief." This concisely and clearly expresses the doctrine I have aimed to inculcate. Extreme skepticism and bigotry are alike inimical to the progress of all true science. The one is too apt to reject; the other too slow to change: the one makes but little progress, because always lost in the wilderness of universal doubt; the other is incompetent to determine the fair and legitimate bearing of testimony, because obstinately and unreasonably wedded to particular dogmas, creeds and opinions- But an easy *credulity*, in matters of science, is even more objectionable than either. It believes all things—adopts the latest fashions—runs the rounds of empiricism—bleeds and purges to-day for everything, and to-morrow gives brandy and beef-steak;—abandons these extremes for steam, or pepper, or electricity, or sugar of milk potentialized by trituration, until the last and latest Vision, like the ideal hero of the Æneid, escapes in a cloud from the scene of action, and leaves its votary to seek for other aids and better lights to guide him.

Personally we do not know Prof. Armor—neverthelefs he excites our deepest anxiety. Fogyism is rampant in his present section, and we warn him that it will never do for him to there enunciate such "paradoxes and dogmas,,' as are contained in the lecture before us. We look for yet another catastrophe at Ann Arbor. There are Dogberrys and Jack Cades all around him. Let him take our warning in time. Let Gunn load himself for another battle, and let both take Sager counsels. He may escape by Ford-ing, but the Palmer (*no* from the Holy Land,) and the Black Douglass will surely pursue him with the bloodhound beagles of the Regency.

---

*A Case of Neuroma of the Optic Nerve, with Remarks and Illustrations.* By John A. Lidell, M. D., Surg. U. S. Vol., Prof. Anat. Nat. Med. College. Second Edition. New York. 1863.

Our thanks are due the author for a copy of this very interesting pamphlet. It describes probably the only case on record of Neuroma of the Optic Nerve, and almost the only one involving a nerve of special sense. It should be borne in mind that not every tumor developed in connection with a nerve is properly neuroma.

We extract from the article a few remarks of a descriptive and diagnostic character regretting that our limits forbid insertion of the details of the case in full, which was permanently cured by extirpation.

These nerve-tumors vary in size, from a pin-head to a melon. In shape they are regular, and either oval or elongated, their long axis always corresponding with the direction or course of the affected nerve. In structure, they are either cystic, which is the exceptional form, or cystico-solid, or entirely solid, (sarcomatous,) the latter being the common form. It is believed by some surgeons that, when the solid growth reaches a considerable size, one or more cysts, or cavities, filled with yellowish, brownish, or reddish-brown liquid, are apt to form in the inteaior, their formation being apparently due to a process of disintegration having commenced therein. Sometimes the tumor appears to be composed of concentric layers. In color, the interior structure varies from white or grayish-white, to a yellowish or reddish-yellow hue. In consistence, they vary from the denseness of fibrous tissue, and the hardness of cartilage, to the soft, fluctuating feel of a sero-cyst. With regard to mobility, *neuromata* are, for the most part, *distinctly movable in the direction of their transverse diameter*, but not in the direction of their long diameter, and this mobility is seldom or never destroyed by contracring adhesions to neighboring parts. Every effort to move these tumors in the course of their long diameter is productive of great pain. The skin investing these morbid growths is never discolored, and the cutaneous veins are never enlarged and tortuous, except the tumors have attained an unusual size; indeed, the integument in general is never affected by this disease in any other way than by mechanical pressure. These nerve-tumors are surrounded or invested with a distinct capsule formed from cellular tissue, condensed by the progressive expansion of the swelling, and it frequently happens that, within this covering, a second can readily be distinguished, formed by the expanded sheath of the parent nerve. *Neuromata never invade the contiguous parts by infiltrating them with their own peculiar substance, and thus impoeing on them their own peculiar structure.* They invade the neighboring parts only by mechanically pushing them away as they expand, and by slowly producing absorption of them from long-continued pressure. *Neuromata never infects the lymphatic glands*, neither in its own neighborhood nor in remote parts of the body, *and when properly excised it never returns.* In all these respects it differs widely from cancer, and is a disease entirely dissimilar thereto in its real character. Neuromatous tumors are commonly found single, though they are occasionally multiple, and in very rare instances, according to the monograph already referred to, as many as several hundred are found in the same subject. The *painful subcutaneous tubercle*, three examples of which are referred to by Chesselden, is one of the most frequent forms of neuroma. Nerve-tumors, though commonly, are not always painful. The simple is much more liable to be painful than the multiple form of the disease. The pain, when present, has a peculiar character; the torture is agonizing, and the pain darts "along the trunk and branches of the nerve, with all the suddenness of an electric shock;" the pain is paroxysmal, and the exacerbations may be produced by pressing upon, handling or jarring the tumor, by strong mental emotion, by fatigue, and by changes in the hygrometrical condition of the atmosphere, disappearing in dry and clear, and coming on again in damp and cloudy weather. In respect to origin, neuroma is considered to be either spontaneous or trumatic; but whether spontaneous or traumatic, we are no nearer the truth with regard to the special perversion of the nutritive function, upon which the growth of such masses depends. Traumatic neuroma is most frequently seen in the stump of amputated limbs, and its treatment should be conducted on precisely the same principles as that of the spontaneous form of the disease.

Neuromata generally increase in size slowly and steadily. It is said that they never retrocede or disappear spontaneously. Medicines, whether applied locally, or taken internally,do not appear to possess the power to stop, or even to retard their growth materially. Expirpation affords the only certain cure, and extirpation by the knife, or excision, is preferable to that effected by any other method. Excision of a portion of the parent nerve, together with the tumor, has also been recommended, but I can not conceive that this procedure is often necessary. Sometimes, by the exercise of great care and skill, the tumor can be dissected away from the nerve with which it is in contact, without dividing said nerve, a feat which Velpeau accomplished in a case of neuroma sciatica. These tumors shauld not be excised, unless they are painful, or cause inconvenience to the patient by their size or weight.

Neuroma is distinguished from carcinoma by the following facts: 1. It never affects the surrounding parts otherwise than by pressure, whereas carcinoma involves the neighboring tissues by infiltrating them, and imposing on them its peculiar structure. 2. It never contaminates the neighboring lymphatic ganglia; carcinoma does. 3. While it impairs the patient's general health by pain and distress, causing pallor, emaciation and debility, it never produces the true cancerous cachexy by specific infection of the system; carcinoma does produce such a result. 4. It does not return after excision; carcinoma does. 5. The pain of neuroma is neuralgic (electrical and paroxysmal) in character; the pain of carcinoma is not.*

---

*Obituary Record.*—It is with deep regret that we find in the *Boston Journal* the announcement of the death, by apoplexy, of our old friend Dr. Geo. Hayward. Dr. H. was one of the oldest and ablest surgeons of Boston, and a most hospitable, public-spirited, and high-minded gentleman. He was always foremost in aiding all useful objects, and his death will be a severe loss to the city of Boston and to the profession of this country.—*Med. News.*

---

*Enterotomy.*—Dr. Reali relates the following case: A countryman of limited capacity had introduced a piece of wood into the rectum, nine days before he applied to the Hospital of Orvieto, complaining of severe pain in the abdomen. On exploration the finger was able only just to reach the point of a piece of wood lying surrounded by the turgid mucous membrane in the direction of the hollow of the sacrum. Numerous attempts were made with various instruments to extract the foreign body without success, and, as the patient suffered greatly, an incision was made into the left iliac region. The finger having been introduced, it was found that the foreign body, having passed into the sigmoid flexure, the latter was distended, and forced to the mesial line, the body it contained rising as high as the navel. The intestinel was incised, and a truncated cone of chestnut wood (irregularly shaped, and measuring twenty-five centimetres in length, and from nine to ten in diameter) was removed. The wound in the intestine was closed by Jobert's procedure, and oily purgatives were administered. Great meteorism, entero-peritonitis, and an iliac abscess were among the sequelæ; but the patient recovered, and remained well when seen two years afterwards.—*Med. Times and Gaz.*

---

*Strangury from Cantharides.*—Dr. Amuille, of Paris, extols the method of treatment introduced by Dr. Mulock, of

*Vide Smith's monograph.

Dublin, for the cure of strangury from cantharides. This consists in giving liquor potassa, in doses of half a drachm, in gruel or flaxseed tea, every hour. Dr. A. says that he never finds more than two or three doses required to effect a cure.—*Med News.*

---

*Mode of Preventing Injurious Action of Lead Pipes on Water.*—The importance of discovering a really efficient means of preventing the injurious action of lead pipes on water is universally acknowledged, and the experiments of Dr. Crace Calvert have proved beyond question that no proposition hitherto brought forward has been calculated to remedy the evil complained of. A discovery, however, has now been made through which the water supplied by leaden pipes may be obtained by the consumer as pure as from the original source. Dr. H. Schwartz, of Breslau, has discovered a means by which the portion of the lead forming the interior surface of the pipe may be converted into an insoluble sulphide, the natural consequence being that the water passing through will be as free from contamination as if glass were used. The means by which Dr. Schwartz effects this conversion are extremely simple. He simply passes a strong solution of the sulphide of an alkali through the pipe to be acted upon, and the process is completed. This solution, which is either a sulphide of potassium or of sodium, is used at a temperature of about 212° F., and is allowed to act upon the metal for from ten to fifteen minutes. It is stated that, in practice, the boiling solution of caustic soda and sulphur is found to answer every purpose.—*Med. Times and Gaz.*, Sept. 26, 1863, from *Mining Journal.*

---

*Sewage.*—Sir Edward Bulwer Lytton made lately the following interesting remarks on this subject.—

"I remember, when I held the Colonial seal, the trouble and toil it cost me to secure from some distant islands a scanty supply of guano, while all the time, close at hand, a few of the London sewers were every year casting away into the Thames more than half a million's worth of a manure considerably more valuable for the general purposes of agriculture than that guano which ships were fitted out to bring home, in order that it might be retailed at a price which rather fits it for the phials of an apothecary than the fields of a farmer. I said half a million's worth of money was thus thrown away, but that is a very low estimate of the real waste. In Flanders, for instance, where I have been lately, the value of sewage is calculated according to the numerical population, especially in towns. It is there calculated at £1 7*s.* a head yearly. In Belgium it is calculated at a still higher rate. So that, if the population of London be taken at 2,000,000, a means of increasing the productive wealth of the country (which, according to the estimate of Flanders, would be worth about £2,700,000) is exclusively devoted to poison the waters of the Thames, and administer gratuitous disease to her Majesty's metropolitan subjects. If we may condescend to take lessons from barbarians, the Chinese may in this respect be our teachers. The rapidity with which the Chinese bring almost any soil into cultivation, and, when brought into cultivation, the enormous crops which they contrive to take from mere handfuls of land, have been the wonder and admiration of travelers. But the great secret of the Chinese is in the utilization of sewage. The proverbial fertility of Belgium is owing, in much, to the same cause. But it is not only the sewage of London which is wasted, but that of all our rural towns; although in them there appears a more impatient desire to remedy acknowledged abuses than seems to be the characteristic of city aldermen and metropolitan boards. When I consider how many populous towns there are in this country, I heartily wish we could send among them a few enlightened Chinese engineers to devise the best practical means by which our townsfolk might be enriched by the manure they could sell, and our farmers enriched by the manure they could buy. But in the meanwhile, until some such scheme is devised and agreed to, we must fall back on our friend the farm-yard dunghill, assisted indeed by various chemical manufactures, but never to such a degree as to supply its place. Professor Liebig is, no doubt, right in considering the chief art of productive husbandry to consist in the skilful application of manure. David Hume tells us, in one of his essays, that all the vast apparatus of our government has ultimately no other object or purpose than the distribution of justice, or, in other words, the support of the twelve judges. So it may be said that all the apparatus of productive husbandry has ultimately no other object or purpose than the distribution of justice to the soil—in other words, the application of that manure which gives back to the soil the nutriment we take from it, or supplies the nourishing properties which Nature had neglected to bestow. Eight hundred years ago there was a very learned dispute whether or not the earth was an animal. We have now discovered that the earth is so far an animal that it requires to be fed, and will not bear to be starved. A remarkable instance of this truth is mentioned, by a celebrated agricultural authority, in some of the Southern States of America—such as Maryland and Virginia. In those States there were large districts of some of the most fertile lands in the world. The crops they yielded were prodigious, but, unluckily, the cultivators neglected the manure; they took from the land the alkalies and salts which they did not replace, and these districts have now become so hopelessly sterile that they have been altogether abandoned as a desert."—*Lancet*, Oct. 25, 1862.

---

*Life in the Atmosphere.*—James Samuelson, Esq., in a paper read before the British Association at their late meeting, remarked that no subject in natural history, except the allied one, the origin of species, had of late excited greater interest in the scientific world than the origin of the lowest types of living beings on the globe; and although the problem was far from being solved, yet the investigations that had accompanied the discussion had already served the useful purpose of throwing new light on the anatomy and life history of the mysterious little forms of which it treated. It was rather with the latter object, than in the expectation of being able to assist in the solution of the general question, that he ventured to lay before the association the results of investigations recently made. He had, for example, taken rags imported from various countries, and shaken the dust from them into distilled water, which he then exposed to the atmosphere; and, after describing generally the character of the living forms he had discovered in this pure water, he stated in detail the forms of life found in each kind of dust, and among these were some new species of Rhizopoda and Infusoria, and an interesting ciliated worm-shaped form, which he believed to be a collection of the larvæ of some other infusoria. The general results of the microscopical examination of these fluids between the 27th of July and 15th of August was as follows: In the dust of Egypt, Japan, Melbourne, and Trieste, life was the most abundant, and the development of the different forms was rapid. In conclusion, he observed that if he was correct in supposing the germs of the living forms that he had described to be present in the dust conveyed by the atmosphere, and in distilled water, it was worthy of notice that these germs retain vitality for a long period—a period of which he could not pretend to define the limit. In his experiments they outlived the heat of a tropical sun, and the dryness of a warm room during the whole winter; but in Dr. Pouchet's case they retained their life 2,000 years, for he obtained his from the pyramids of Egypt, and then survived an oil of 400 degrees of heat. A main purpose which Mr. Samuelson had in view was to disprove the theory of spontaneous generation; and he suggested whether the great rapidity with which these germs are multiplied might not account for the spread of epidemic diseases. He did not profess to have any acquaintance with such diseases, but might it not be desirable to subject the atmosphere of hospitals to the microscopic test?—*Med. Times and Gaz.*, Oct. 3, 1863.

---

*Obituary.*—Charles P. Marsh, M. D., died at Kalamazoo, Mich., Nov. 17th, 1863, Æ. 35. Dr. Marsh graduated at the Med. Dep. of the Univ. of Mich. in the spring of 1851, and in the twelve years of his subsequent engagement in the practice of his profession, he had a variety and extent of experience which very rarely falls to the lot of a private practitioner. With his brother, Wells R. Marsh, M. D., now a Medical Director in the army, he was solicited, soon after graduating, to take the medical charge of the colony of Hollanders, established in the region of Black Lake, Ottawa Co., Mich., under the auspices of the Rev. A. C. VanRaalte. Some six thousand or more of these emigrants, mainly of the better class, were in a few months removed from the old country and located in the dense forests, as yet untouched by the axe, but which the far-seeing and sagacious pioneers saw contained all the elements of future prosperity.

Speedily mastering the language with great perfection, Dr. M., by his rare skill in diagnosis and great practical tact in treatment, combined with most untiring devotion to his duties and warmly sympathetic feelings, endeared himself most remarkably to the intelligent and industrious community among whom his lot was cast.

It is noteworthy that in his practice (and the whole burden soon fell upon him, in consequence of the appointment of his brother as Professor in one of our prominent Western Colleges,) there was scarcely an operation in Surgery or Obstetrics which it did not fall to his lot to perform, often most unexpectedly, and with the crudest appliances. Genius supplied the place of delicate instruments and costly apparatus. He extemporized means, and success crowned his operations in the most striking degree. After several years of labors, under which the most iron constitution could not fail to succumb, his health failed, and a chronic diarrhœa supervening upon a severe course of Typhoid Fever, ultimately compelled him to abandon the field of his arduous practice.

Soon after this, although still suffering unner the effects of his disease, he became connected with the army, commencing his services on the memorable Sunday at Pittsburg Landing, afterwards upon the Hospital boat, City of Memphis, and then in connection with the 44th Ill. Infantry. In all these relations he acquitted himself with credit to himself and advan tage to the service. No heart was more open to sympathy, no hand more firm in its work. No toil or watching dismayed him. Many a soldier's grateful prayers will throw a halo around his memory.

From these duties he was suddenly recalled to attend as it was supposed the last hours of a fondly beloved father. While the father however yet lives, the son has passed from earth, lamented by his humble friends the Holland emigrants as a dearly beloved benefactor; by those of our diseased and wounded soldiery who had had the benefit of his pains-taking, tireless skill, as a conscientious, self-sacrificing patriot; by those who knew him in private life as the genial companion, the amiable gentleman, the honest, noble-souled man. A constitution enfeebled by chronic, almost uncontrollable, disease, was broken wholly down in a few hours by *Pneumonia Typhoides.* After life's fitful fever he sleeps well. Peace to his ashes.

*Hospital Gangrene, etc.*—In the next No. of the Journal we shall notice at length the exceedingly interesting report to the Surgeon-General, of Prof. MIDDLETON GOLDSMITH, Surgeon U. S. V., Louisville, Ky., on the use of Bromine, etc., in Hospital Gangrene, Erysipelas, and Pyæmia, as observed in the Depts. of the Ohio and Cumberland. We acknowledge its receipt with unmixed pleasure.

Our next No. will also contain an elaborate paper on the same subject, from an intelligent correspondent connected with the U. S. Hospitals in Memphis, Tenn.

The subject is one of peculiar importance and interest at the present time, and we welcome to our pages all earnest, thorough and clear-sighted examinations of the principles, facts and agencies involved.

---

*Open Air Treatment of Phthisis.*—Dr. Blake concludes another of his valuable treatises on the open air treatment of Phthisis:

Unfortunately but few localities afford facilities for fully carrying out this plan of treatment. Certainly in no other part of the globe can it be more readily tried than on this coast. For six months in the year we have a rainless climate, during the whole of which time our patients can live in the open air, under the most favorable hygienic conditions, and when the rainy season here would drive them into houses, a sea voyage of four or five days brings them to Northern Mexico, where, during the winter months they can enjoy a climate exactly analogous to our summer climate here, as in that country rain only falls between June and October. The intervening months are absolutely rainless. I have seen cases of consumption, in which a winter's residence there has been most beneficial, and I have a patient wintering there at present, who is reported as improving rapidly, although the disease had advanced to the third stage. When our patients are in a position to avail themselves of the advantages of the summer climate of California, and of the winter climate of Mexico, I believe there are few cases, except those in which the destruction of lung tissue is already too extensive, that cannot be cured. Many cases, both in the first and second stages, can be cured by merely passing the summer out of doors in this country, but even in these, I believe wintering in Mexico would very much shorten the treatment. In all cases that have advanced to the third stage, wintering in Mexico should be recommended, as the treatment is much prolonged by the aggravation of the symptoms, during the time that the open air treatment has to be suspended. Every case, however, must be treated on its own merits; the time and means required for restoration to health, being more dependent on the general pathological and diathetic condition of the patient, than on the amount of change that has taken place in the lungs.

It is not my intention at present to enter on the questions of pathology and treatment which the above facts naturally suggest. This I hope to do on some future occasion. Their most important bearing I believe is, the support they bring to the views of those who place their chief relianee on the hygienic treatment of the disease. How striking a confirmation do these facts offer of the remark of Dr. Richardson, who, in his work on Consumption, observes:—

"In a cozy room the consumptive is bound never to live, nor in any room indeed, for any great length of time. So long as he is able to be out of doors, he is in his best and safest home. In the fields, on the hills, wherever the fresh air vivifies, where plants look most vigorous, and animals frisk about in the joy of health, there will the consumptive draw in his choicest medicine, there dissolve and throw off most freely the germs of his disease, and there repair most easily the tissues he has lost."

---

Received from the Publishers, by John R. Walsh, Elmir's Hand-Bood of Practice. This convenient pocket memorandum for practicing physicians is for sale by all our principal booksellers.

---

ERRATA.—Page 415—5th line from bottom, for Aqueduct read Viaduct.
Page 416—12th line from top, for Ecrassum read Ecraseur.
" " —14th line from bottom, for Paul Dubois read M. Depaul.
" 418—8th line from top, for Lithotomy read Lithotrity.

---

VACCINE MATTER.—Carefully selected Vaccine Matter may be obtained by enclosing One Dollar and addressing *Chicago Medical Journal*, P. O. Drawer 5787, Chicago, Ills.

